# Exceptional in vivo catabolism of neurodegeneration-related aggregates

**DOI:** 10.1186/s40478-018-0507-3

**Published:** 2018-01-29

**Authors:** Zsolt Datki, Zita Olah, Tibor Hortobagyi, Lilla Macsai, Katalin Zsuga, Livia Fulop, Zsolt Bozso, Bence Galik, Eva Acs, Angela Foldi, Amanda Szarvas, Janos Kalman

**Affiliations:** 1 Department of Psychiatry, Faculty of Medicine, University of Szeged, Kalvaria sgt. 57, Szeged, H-6725 Hungary; 2 MTA-DE Cerebrovascular and Neurodegenerative Research Group, Department of Neuropathology, Institute of Pathology, University of Debrecen, P.O. Box 24, Debrecen, H-4012 Hungary; 3 Department of Old Age Psychiatry, Institute of Psychiatry Psychology & Neuroscience, King’s College London, Box PO70, De Crespigny Park, Denmark Hill, London, SE5 8AF UK; 4 Agrint Kft., Facan sor 56, Godollo, H-2100 Hungary; 5 Department of Medical Chemistry, Faculty of Medicine, University of Szeged, Semmelweis u. 6, Szeged, H-6725 Hungary; 6 Bioinformatics & Scientific Computing, Vienna Biocentre Core Facilities, Dr. Bohr-Gasse 3, 1030 Vienna, Austria; 7 Danube Research Institute, MTA Centre for Ecological Research, Karolina ut 29-31, Budapest, H-1113 Hungary; 8 Sustainable Ecosystems Group, MTA Centre for Ecological Research, Klebelsberg Kuno u. 3, Tihany, H-8237 Hungary

**Keywords:** Alpha-synuclein, Bdelloid rotifer, Beta-amyloid, Catabolism, Lifespan, Prion

## Abstract

Neurodegenerative diseases are linked to a systemic enzyme resistance of toxic aggregated molecules and their pathological consequences. This paper presents a unique phenomenon that *Philodina acuticornis*, a bdelloid rotifer, is able to catabolize different types of neurotoxic peptide and protein aggregates (such as beta-amyloids /Aβ/, alpha-synuclein, and prion) without suffering any damage. *P. acuticornis* is capable of using these aggregates as an exclusive energy source (i.e., as ‘food’, identified in the digestive system and body) in a hermetically isolated microdrop environment, increasing their survival. As regards Aβ1–42, five other bdelloid rotifer species were also found to be able to perform this phenomenon. Based on our experiments, the Aβ1–42-treated bdelloid rotifers demonstrate significantly increased survival (e.g. mean lifespan = 51 ± 2.71 days) compared to their untreated controls (e.g. mean lifespan = 14 ± 2.29 days), with similar improvements in a variety of phenotypic characteristics. To our knowledge, no other animal species have so far been reported to have a similar capability. For all other microscopic species tested, including monogonant rotifers and non-rotifers, the treatment with Aβ1–42 aggregates proved to be either toxic or simply ineffective. This paper describes and proves the existence of an unprecedented in vivo catabolic capability of neurotoxic aggregates by bdelloid rotifers, with special focus on *P. acuticornis*. Our results may provide the basis for a new preclinical perspective on therapeutic research in human neurodegenerative diseases.

## Introduction

Neurodegenerative disorders, such as Alzheimer’s disease (AD), Parkinson’s disease (PD), and prion disease, could be regarded as phenotypes secondary to the progressive functional impairment of proteomes. [29, 52, 53]. The molecular basis of aging in the brain may be described as an accelerated accumulation accompanied by a decreased clearance and degradation of misfolded proteins [46]. There is a clear correlation between protein aggregation and aged-related pathologies. The intramolecular regions arranged in β-sheet conformation are highly resistant to enzymatic degradation. [32, 51] The various neurotoxic aggregates, such as those composed of beta-amyloid (Aβ), alpha-synuclein (α-Syn), and prion (normal cellular prion protein /PrPC/ and pathogenic prion protein ‘scrapie’ /PrPSc/), share common features, with their accumulation and aggregation facilitating neurodegeneration. The peptide and protein aggregates in neurodegenerative diseases have several characteristics in common; however, their different molecular structures and pathomechanism may lead to differences in their toxicity [38]. Therefore, investigation of aggregate degradation has emerged from a marginal area of protein chemistry to become a highly relevant field in neuropharmacological science [25]. Although the pathological role of these aggregates has been well established, at present, no universal and satisfactory method exists for their in vivo degradation as a potential therapeutic tool. Misfolded peptide and protein aggregates can be partially digested by several endogenous enzymes, such as insulin-degrading enzyme (IDE) [23], neprilysin (NEP) [16], endothelin-converting enzyme [12], angiotensin-converting enzyme [14], plasmin [47] and matrix metalloproteinases [1]; however, their presence and function is apparently insufficient in a scenario that leads to neurodegenerative disorders.

Amyloids, such as Aβs, are key molecules in aging-associated diseases, representing a starting point in the development of dementias. Therefore, their accumulation is one of the most important toxic processes during the course of cerebral Aβ-related pathologies, which is potentiated by a reduced clearance and insufficient degradation [30]. The understanding and modulation of Aβ toxicity and its metabolism might provide novel approaches in the treatment of Aβ-related dementias, including AD and cerebral amyloid angiopathy.

Physiologically, two major enzymes are predominantly implicated in the partial degradation of Aβs: NEP and IDE [6, 16]. NEP is a membrane-anchored zinc-dependent endopeptidase, being able to cleave both Aβ monomers and oligomers. The role of NEP in the pathogenesis of AD is indicated by its decreased expression in the AD brain, particularly in vulnerable regions such as the hippocampus and the midtemporal gyrus, a phenomenon associated with increased Aβ-deposition [54]. IDE, a thiol- and zinc-dependent metallopeptidase, appears to participate in the catabolism of insulin and Aβ as well, and its decreased expression was reported in the hippocampus of AD patients [55]. Although IDE mediates these processes in vivo*,* it still remains a question how this cytoplasmic protein can degrade extracellular Aβ aggregates in the brain. Further relevant membrane proteases involved in Aβ degradation include plasmin, cathepsin B, endothelin-converting enzyme, and certain members of matrix metalloproteinase family, which are highly tissue- and brain region-specific [2]. Potential therapeutic approaches to reduce the accumulation of harmful neurotoxic proteins include the facilitation of anti-aggregation processes or the enhancement of their clearance. As an example, β-sheet breakers bind to the central hydrophobic core of Aβ1–42 and attenuate the formation of the β-sheet structures. These molecules could destabilize the senile plaques; however, they do not provide adequate solution to the degradation and catabolism of overexpressed toxic aggregates. [40] Therefore, an ideal protective strategy against aggregate-induced neuronal damage requires more complex and practical solutions, with dual mechanisms of action targeting both the destabilization and degradation of toxic aggregates.

Treatments with different exogenous Aβ isoforms are widely used models of AD and earlier studies used various in vitro and in vivo systems to reveal their exact effects. Several studies were performed on human neuroblastoma cells [7, 36], invertebrates, rodents, and primates [13, 20, 42]; however, only a single publication aimed at describing the effects of Aβ on bdelloid rotifers, e.g. *Philodina* species [36]. This unique study by Poeggeler et al. [36] reported the treatment of rotifers with Aβ1–42 in order to test the efficacy of an antioxidant molecule (LPBNAH) against the supposed neurotoxicity of the peptide aggregates. In their in vivo studies with rotifers, the authors applied doxorubicin instead of Aβ1–42, because this toxin gave more consistent results in rotifers. In fact, the neurotoxic effect of Aβ1–42 in this model could not be proven. Our aim was to investigate this intriguing phenomenon that was only slightly touched upon in the paper of Poeggeler.

Bdelloid rotifers, as microinvertebrates, are one of the most commonly used animal models in toxicity-, aging-, and longevity-related research. These organisms are multicellular animals with well-defined anatomical characteristics, possessing a ciliated head structure, bilateral ovaries, mastax, ganglia, muscles, digestive, nervous, and secretory systems, and photosensitive, and tactile organs. [5, 15]. Due to their peculiar anatomy and physiology, these animals have outstanding advantages in terms of culturing and are rather easy to work with [44]. Rotifers are extremely resistant to environmental alterations and successfully adapt to the different types and amounts of nutrients present in their natural habitat. The natural decomposition of organic materials is a process that results in the formation of precipitates and aggregates, which represent potential nutrients for rotifers [50]. The metabolic utilization of all these available organic material resources is their special property [4].

In a prior publication, we reported the development of a unique and straightforward method [34], which enables the investigation of the effect of several different agents or impacts on various phenotypic parameters of microinvertebrates. The oil-covered microdrop technology, adopted from human in vitro fertilization, is a well-controllable construction to assess the lifespan and other features of a single isolated animal (one-housed rotifer).

In our present study, we examined the effect of various neurodegeneration-related peptide and protein aggregates under complete dietary restriction, ensuring that the individual rotifers had no other organic source to be used for gluconeogenesis. Observing an intriguing increase in survival upon treatment with aggregates, as a next step, we investigated different types of micro-entities in neurotoxic aggregate-supplemented environment. To our knowledge, this study is the first to address the in vivo catabolism of these molecules as dietary sources in microscopic animals such as rotifers. Our findings may provide a starting point to understand the possible ways of degradation of abnormally folded neurotoxins despite their aggregated state and consequent protease resistance, a subject with high potential relevance in the treatment of neurodegenerative proteinopathies.

## Materials and methods

### Materials

The Aβ1–42, Aβ1–42 [Gln22], Aβ1–40, Aβ25–35, two scrambled isoforms (Aβ1–42 S1: LKAFDIGVEYNKVGEGFAISHGVAHLDVSMFGEIGRVDVHQA and Aβ1–42 S2: KVKGLIDGAHIGDLVYEFMDSNSAIFREGVGAGHVHVAQVEF) were prepared in the Department of Medical Chemistry, University of Szeged, Szeged, Hungary. The peptides were synthesized on an Fmoc-Ala-Wang resin using Nα-Fmoc-protected amino acids with a CEM Liberty microwave peptide synthesizer (Matthews, NC, USA). The peptide Aβ11–42 (H-7668.1000) was purchased from Bachem (Torrance, CA, USA), whereas Aβ1–28 (A0184) and α-Syn (type E46K human; S4447) were purchased from Sigma-Aldrich (St. Louis, MO, USA). The mature part (25–244) of recombinant bovine prion protein (PrPC, AG210) was obtained from Merck Millipore (Darmstadt, Germany). EZ4U (BI-5000; Biomedica Medizinprodukte, Wien, Austria) and Calcein-AM (17,783; Sigma-Aldrich) cell viability assays were used to measure the toxicity of the aggregates. For in vivo and in vitro investigations of the different aggregates, we applied Bis-ANS (4,4′-dianilino-1,1′-binaphthyl-5,5′-disulfonic acid dipotassium salt; D4162) and Congo red (CR; C6277) dyes obtained from Sigma-Aldrich. To detect gold-tagged beta-amyloid (Au-Aβ1–42) in *P. acuticornis* with scanning electron microscopy (SEM), we used Gold(III) chloride (AuCl3 x 2H_2_O; 01216, Reanal, Budapest, Hungary) and Aβ1–42 aggregates. Distilled water (DW) was prepared in our laboratory (Millipore-type, ultrapure, demineralized DW).

### Preparation of aggregating peptides and proteins

The synthesis and characterization of the Aβ peptides were conducted as previously described by Bozso et al. [3] with minor modifications: the concentrations of the stock solutions were 1 mg/mL (DW); the aggregation time was 3 h or 3 days (25 °C, pH 3.5); the neutralization (to pH 7.5) was performed with NaOH (1 N) [17]; after 10-fold dilution with standard medium, the final (working) concentrations were 100 μg/mL. The amount of diluted cations and anions in standard medium (mg/L): Ca^2+^ 31.05; Mg^2+^ 17.6; Na^+^ 0.9; K^+^ 0.25; Fe^2+^ 0.001; HCO_3_^−^ 153.097; SO_4_^−^ 3; Cl^−^ 0.8; F^−^ 0.02; H_2_SiO_3_ 3.3 (pH = 7.5) [41]. To prepare the PrPSc form of PrPC, the stock solution of PrPC was aggregated for 24 h at pH 2 [49, 57]. The pH of the prepared prion was also adjusted to pH 7.5 before being used to treat the rotifers.

### Collection, isolation, identification and harvesting of different animal species

To collect and isolate different microscopic species, we used the method described by Debortoli et al. [9] with minor modifications. The sampling sites were distributed within two areas of about 500 m^2^ near Szeged (Southern Great Plain, Hungary) and Saint-George (Transylvania, Romania). Fifty patches of highly hydrated moss (with minimal soil) were collected from the northern side of trees (of the genera Acer and Platanus) and from their close environment. Briefly, the collected samples were hydrated with the standard medium in separate flasks. After identifying the species by using methods described in the literature [18, 21, 24, 33, 48] we applied species-specific information (e.g., body size in relation to age) to collect relatively young (approximately 3–5 days old) and individuals of the same size. The isolation protocol from the samples was in line with the method of Debortoli et al. [9]. However, instead of culturing; we collected and isolated these animals (using micropipette) to create monoclonal populations. After washing these groups with standard medium, we harvested as many individuals as needed (*n* = 30) for the experiments. The species collected in Hungary comprised *Philodina vorax Janson, 1893; Philodina megalotrocha Ehrenberg, 1832; Lepadella patella Müller, 1773; Lecane arcula Harring, 1914; Lecane agilis Bryce, 1892; Lecane hamata Stokes, 1896; Lecane closterocerca Schmarda, 1859; Brachionus diversicornis Daday, 1883; Brachionus calyciflorus calyciflorus Pallas, 1766; Brachionus forficula Wierzejski, 1891; Caenorhabditis elegans Maupas, 1900; Typhloplana viridata Abildgaard, 1789; Vorticella convallaria Linnaeus, 1758; Stylonychia mytilus Müller, 1773*. The species collected in Romania comprised *Mniobia russeola Zelinka, 1891; Adineta steineri Bartoŝ, 1951; Habrotrocha elusa Milne, 1916; Filinia terminalis Plate, 1886; Lindia torulosa Dujardin, 1841; Hypsibius dujardini Doyère, 1840; Centropyxis aculeata Ehrenberg, 1832.* Processes related to culturing and harvesting *Philodina acuticornis Murray, 1906* were performed (in artificial environment) as previously described by our group [34]. The animals were photographed (Nikon D5500, Nikon Corp., Kanagawa, Japan) under the light microscope (at 200× magnification; Leitz Labovert FS, Wetzlar, Germany) by taking serial images at every 5-μm intervals, yielding a total of 20–30 photographic layers per animal, which were subsequently merged into one superimposed picture (by using Photoshop CC software, Adobe Systems Inc., San Jose, CA, USA) to achieve better resolution.

### In vitro and in vivo treatment and monitoring

To test the previously described neurotoxic effects of the examined aggregates in vitro*,* we used a differentiated SH-SY5Y human neuroblastoma cell line (Sigma-Aldrich), with the related culturing and differentiating methods based completely on our previous works [7, 26]. Five-day-old animals (as determined by BSI) were chosen for the experiments, an age that precedes the beginning of the reproductive stage (i.e., egg production) by 2 days. Before treatment (using micropipette) of individual rotifers with aggregated molecules (0.1, 10 and 100 μg/mL), the stock solutions (1 mg/mL) were ultrasonicated (Emmi-40 HC, EMAG AG, Mörfelden-Walldorf, Germany) for 10 min at 45 kHz to achieve sterilization and homogenization. During the in vivo experiments, the viability assay, the assessments of descriptive characteristics (such as normalized mean lifespan /NML/, body size index /BSI/, bright light disturbance /BLD/, mastax contraction frequency /MCF/, and cellular reduction capacity /CRC/), and the assembly of the experimental setup (with the oil-covered microdrop) were carried out as presented in detail previously [34].

### Detections of exo- and endogenic Aβ1–42 in *P. acuticornis*

**Fig. 1:**
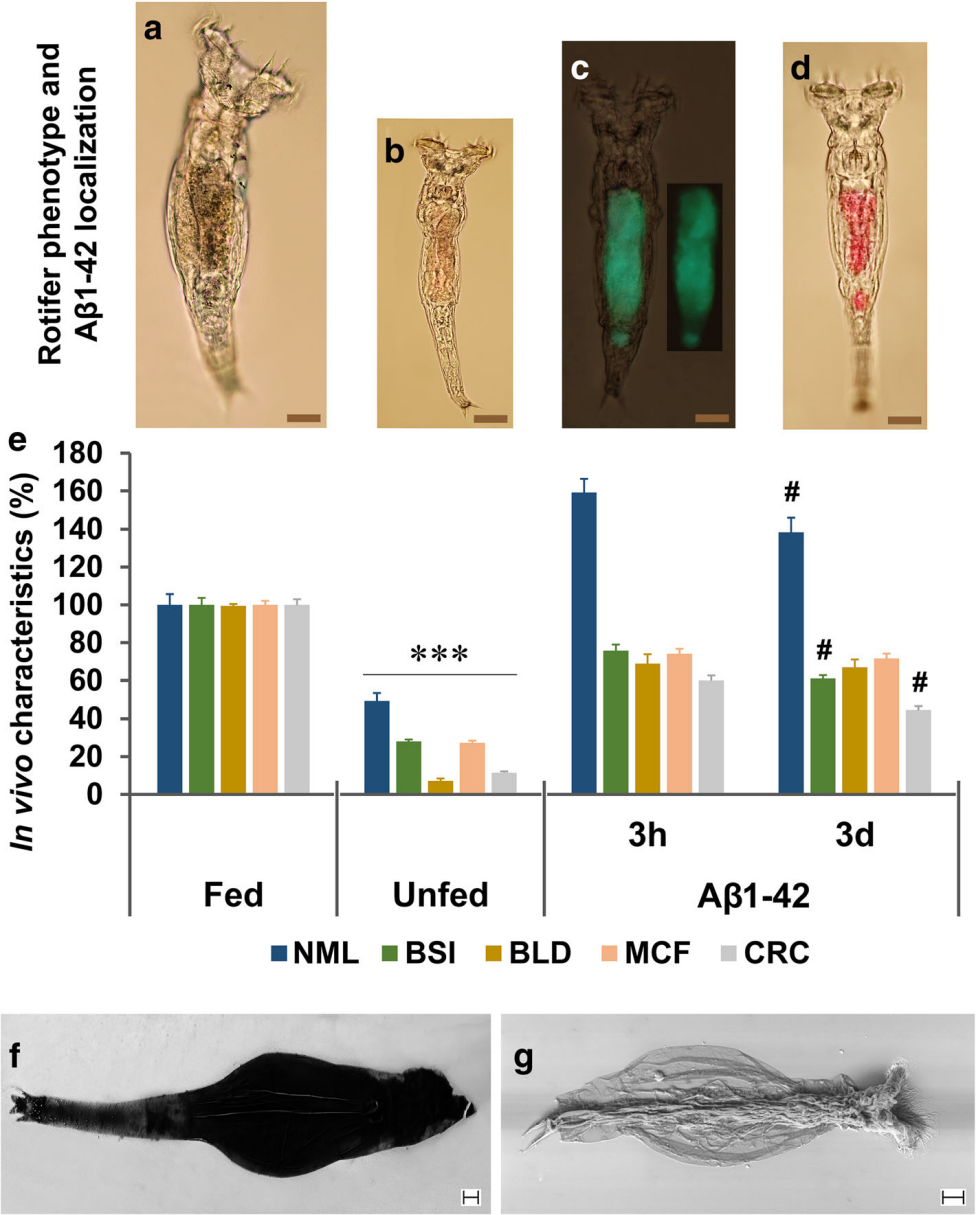
Localization and demonstration of the beneficial effects of aggregated Aβ1–42 on *P. acuticornis.* Juvenile rotifers were selected in different groups after 1-day starvation: fed (**a**; 12 days normal feeding), unfed (**b**; 12 days starvation) and Aβ1–42 treated (for 12 days). The Aβ1–42 in the digestive system (stomach and intestine) of live rotifers were visualized by fluorescent Bis-ANS (**c**; green colour in the representative photograph; 3 h = aggregated for 3 h) and absorbent Congo red (**d**; red colour in the representative photograph; 3d = aggregated for 3 days). Untreated individuals labelled with these two dyes showed no signal (not shown). **e** The normalized mean lifespan (NML) of the Aβ1–42-treated animals was significantly longer than that of their normally fed and unfed untreated controls, with a significant difference between the 3 h- and 3d–aggregated forms. Besides NML, four viability markers were measured, including body size index (BSI), bright light disturbance (BLD), mastax contraction frequency (MCF), and cellular reduction capacity (CRC). The Aβ1–42-treated rotifers performed significantly better than their unfed untreated controls and there were significant differences between the subgroups treated with two the differently aggregated forms in BSI and CRC. The concentration of Aβ1–42 was 100 μg/mL; *n* = 30 (NML), 50 (BSI), 20 (BLD), and 24 (MCF) one-housed individual rotifers per group; *n* = 24, well with normalized absorbance to rotifer number (CRC). The scale bars in the proportional representative photographs represent 20 μm (*, significant difference from the fed and Aβ1–42-treated groups; #, significant difference from the group treated with the 3 h aggregated form). The possible distribution of the exogenous Au-Aβ1–42 complex was studied in vivo with SEM in the body of *P. acuticornis*: **f** rotifer without treatment (background); **g** Au-Aβ1–42-treated rotifer. Homogenously signals of gold can be detected in Au-Aβ1–42-treated entities. Scale bar represents 10 μm

*Optical analysis in live rotifers* (Fig. 1a-d): The juvenile (5 days old) animals were identified on the basis of their body size as read from a calibration curve previously described [34]. For the treatment (in 96-well plate; 3695, Costar, Corning Inc., USA) of preselected and 1-day starved rotifers, we used unlabelled Aβ1–42 (3 h and 3 days aggregated) as ‘food’ source. After 12 days the content of the wells were changed to labelled Aβ1–42, aggregated for 3 h (in vitro marked with 10 μM Bis-ANS fluorescent dye for 30 min) or 3 days (in vitro marked with 50 μM CR dye for 1 h). Applying 5 h treatment (‘feeding’), we detected the optical signals (at 200× magnification) in the digestive system (stomach and intestine) of individual rotifers by an optical/fluorescence microscope (Olympus IX71, OLYMPUS, Budapest, Hungary). The representative images demonstrate the localization of Aβ1–42 in the body of rotifers compared to untreated, normally fed (600 μg/mL yeast suspension) and unfed (starved) controls (Fig. 1a-d). Unfed individuals labelled with these two dyes (control background without Aβ1–42 treatment) have no signal (photo not shown).

*Searching for endogenous Aβ1–42 in rotifers*: The immunological detection of the presence or absence of natural Aβ1–42 in *P. acuticornis* species has a central role in interpreting the relations of Aβ1–42 and rotifers. After harvesting, the animals were put into − 75 °C for 30 min in standard medium. The number of rotifers were 10^4^ per sample. The thawed animals were ultrasonicated (at 45 kHz at room temperature for 10 min). The rotifer homogenate was centrifuged (300 x g for 5 min at 24 °C) to eliminate the exoskeletons of the dead animals. The protein concentration of the supernatant was determined by the Qubit Protein Assay Kit (Q33212; Thermo Fisher Scientific, Wilmington, DE, USA) following the manufacturer’s protocol. The concentration of the stock suspension was 340.54 μg/mL, which was diluted to the final protein concentrations (μg/mL) of 20, 15, 10, 5 and 1 in standard medium. Commercially available Aβ1–42-specific sandwich ELISA kits (Innotest Aβ1–42, 81,576, Fujirebio, Gent, Belgium) were used according to the manufacturer’s protocol for the quantitative detection of Aβ1–42 in the diluted rotifer homogenate.

*The Au-Aβ1–42 complex detection with SEM* (Fig. 1f, g): The stock solutions of Aβ1–42 (1 mg/mL) and AuCl_3_ (2.8 mg/mL) were prepared with DW. The 3-day-aggregated Aβ1–42 solution was mixed for 2 h with the AuCl_3_ solution in a 1:4 M ratio, according to the number of Au-binding sites of monomeric Aβ1–42 (one methionine and three histidine residues). To remove excess Au ions, two rounds of centrifugation (25,000 x g for 10 min at 24 °C) with supernatant replacement were used. NaOH (1 N) was applied to adjust the pH to 7.5. Peptide content of the final pellet was determined by the Qubit™ Protein Assay Kit (Thermo Fisher Scientific). The middle-aged (15 days old) harvested rotifers were starved (i.e., complete food deprivation) for 2 days. After washing the rotifers in 6-well plates (containing 10^4^ adherent animals per well), each well was treated with Au-Aβ1–42 complex in a dose of 100 μg/mL for 1 day. The wells were decanted and washed twice with standard medium and after an incubation period of 6 h (the time to empty the digestive tract of the animals), the wells were washed again and the populations were fixed and dehydrated with 96% EtOH at − 75 °C for 5 min, followed by a partial rehydration with 30% EtOH at room temperature for 30 min. After fixation with 1% paraformaldehyde (for 30 min), the wells were washed twice with DW. The collected animals were transferred with pipette to the centre of a round glass coverslip (diameter: 12 mm, thickness: 0.15 mm; 89,167–106, VWR International, Houston, TX, USA), and were allowed to dry. The samples were not coated with gold. The structural integrity of the rotifer bodies was controlled with digitally recorded photographs (Nikon D5500). The selected bodies (based on their quality) were subjected to SEM (Zeiss EVO MA 10, Carl Zeiss, Oberkochen, Germany) [2]. The sample-carrier coverslip was fixed onto a stub using a double-sided carbon tape. The fine structure of the rotifers was observed and photo-documented with the SEM (Fig. 1f, g), operating at 10 kV with an 8-mm working distance, using a backscattered electron detector in variable pressure mode at 30 Pa. The white balance of SEM photographs was normalized.

### Congo red aggregation assay

The methods described by Klunk et al. [19] and Datki et al. [8] were used for these experiments. The preparation and aggregation of the peptides and proteins for the CR assay was the same as described in the in vitro and in vivo treatment protocols. A volume of 10 μL CR stock solution (0.5 mM) was added to each aggregate-containing tube (490 μL; 100 μg/mL) and these mixtures were incubated for 20 min at room temperature and shaken every 5 min (at 50 rpm for 10 s), followed by centrifugation at 25,000 x g for 15 min. The supernatant was carefully removed with a pipette and the sediment (pellet) was resuspended in standard medium (0.5 mL). The aggregate-bound CR content of the suspensions was measured spectrophotometrically at 540 nm with a BMG NOVOStar plate reader (BMG Labtech, Ortenberg, Germany), using a 96-well plate (Costar).

### Statistics

The error bars represent the standard error of the mean (S.E.M.). For comparative statistical analysis, the one-way ANOVA was used followed by the Bonferroni post hoc test with SPSS 23.0 software for Windows. A *p* ≤ 0.05 was regarded as statistically significant, with the different levels of significance indicated as follows: p^#^ ≤ 0.05, p** ≤ 0.01 and p*** ≤ 0.001. Kaplan-Meier curves were applied to present the survival of the groups. The GraphPad Prism 7.0b software (GraphPad Software Inc., La Jolla, CA, USA) was used for the illustration and statistical analysis (log-rank; Mantel-Cox) of survival.

## Results

To investigate the background of less ‘consistent results’ with Aβ1–42 toxicity in bdelloid rotifers reported by Poeggeler et al. [36], we investigated the effect of different neurodegeneration-related protein aggregates on one-housed *P. acuticornis* in an experimental setup [34] inspired by that applied human in vitro fertilization (i.e., oil-covered microdrop). This assay system allows the observation of our model organisms at an individual level. First, we examined the effect of Aβ1–42, which we predicted to be toxic to *P. acuticornis*. Surprisingly; however, treatment of the animals with Aβ1–42 resulted in a significantly longer mean lifespan (51 ± 2.71 days) than in the case of unfed (14 ± 2.29 days) and normally fed (32 ± 2.72 days) controls. To localize and demonstrate the presence of Aβ1–42 aggregates in the body of the rotifers, we used β-sheet-specific fluorescent and absorbent dyes. Animals in the representative photographs (Fig. 1a-d) are shown in proportional sizes and display the strong differences between the groups. The Fig. 1c, d show the presence of the exogenous Aβ1–42 in the digestive system of the rotifers after ‘feeding’ ad libitum (above is the stomach and below is the intestine). To characterize the Aβ1–42-treated *P. acuticornis* animals, we applied some previously published [34] experimental monitoring assay**s**. The results (Fig. 1e) are evidences to the fact that this bdelloid rotifer can use the Aβ1–42 as food in isolated environment without the presence of any other organic material. The NML of groups treated with either 3 h or 3 days aggregated Aβ1–42 significantly increased compared to unfed (starved) controls. The BSI and the BLD indicated phenotypical and physiological changes of the treated animals. These characteristics were increased by 40% and 60% compared to untreated starved controls, respectively. The MCF and the CRC suggested intensified energy level as represented by neuromuscular and cellular-redox activities. These two markers were increased by 46% and 42% compared to unfed entities, correspondingly. The Aβ1–42-treated one-housed rotifer individuals performed much better in the measured parameters than their unfed controls, and they do not drastically differ from the normally fed counterparts. These results suggest that Aβ1–42 is not toxic to *P. acuticornis* and it could be used by them as an exclusive dietary source to live and develop in an hermetically-isolated environment. As the next step, we aimed at detecting the possible presence of endogenous Aβ1–42 in *P. acuticornis* species. We applied ELISA for the quantitative analysis of Aβ1–42 in the rotifer homogenates. Interestingly, our findings indicate that endogenous Aβ1–42 is practically absent in *P. acuticornis* species*,* an observation first reported in the literature. To localize exogenous Aβ1–42 aggregates in the live (Fig. 1a) rotifers (beyond the digestive system) we applied Au-tagged Aβ1–42 aggregates, detected with SEM (Fig. 1f, g). After fixating and drying the Au-Aβ1–42-treated and untreated animals, we monitored the possible distribution of the remnants of the potentially catabolized peptide. We found that in Au-Aβ1–42-treated animals, the signal of gold-ions could be found homogeneously everywhere in their body in contrast to the untreated ones. The only possible source of gold in the samples was the Au-Aβ1–42 complex taken up during life, as no aspecific gold coating was applied. These SEM photos are only representative.

**Fig. 2:**
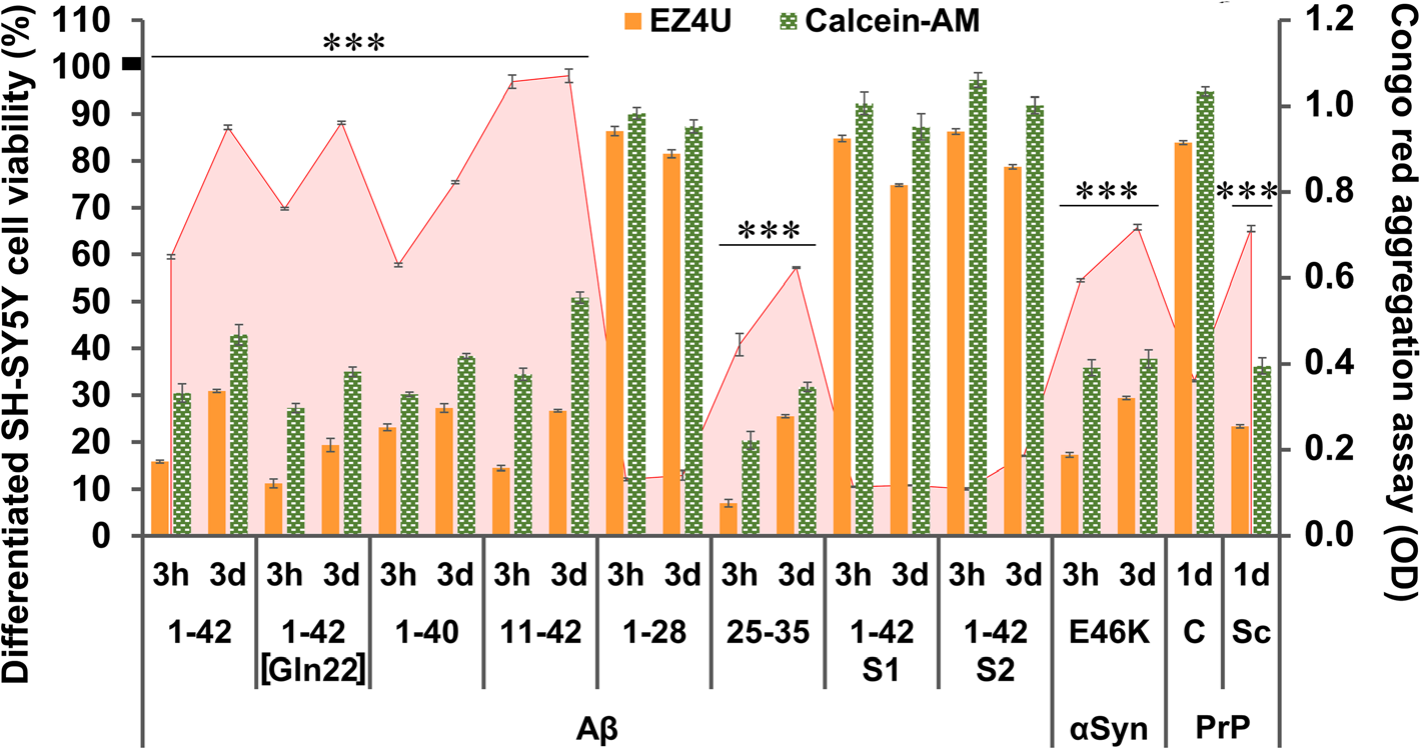
Aggregation-dependent neurotoxicity of different neurodegeneration-related peptides and proteins in cell culture. To test the previously described [10, 11] toxic effect of neurodegeneration-related aggregates, we used a differentiated SH-SY5Y human neuroblastoma cell model. The EZ4U and Calcein-AM cell viability assays were used to detect the NADH- and esterase-activity-dependent cell viability of the cultures (presented in orange and green columns of the chart, respectively). The mean viability of the untreated control wells was regarded as 100% (the S.E.M. of the mean was ±2.8%). The aggregation level of the proteins was measured with Congo red spectrophotometric assay (red line in the background of the chart). The length of incubation and aggregation time (3 h = 3 h and 3d = 3 days) influenced the toxicity of the treatment in most of the peptides and proteins tested. Significant differences (*p* ≤ 0.01) were detected between the 3 h- and 3d–groups (no markers on columns) in both viability assays. Significant differences could be detected between the 3 h- and 3d–aggregated forms in the Congo red assay for all Aβs, except for Aβ11–42, Aβ1–28, Aβ1–42 S1, and Aβ1–42 S2. Alpha-synuclein (α-Syn, type E46K) and prepared ‘scrapie’ (PrPSc) form of prion protein also showed significant in vitro toxicity, contrasting with the ineffective normal cellular prion (PrPC). The aggregation time of the prions was 1 day (1d). Each molecule was used in 100 μg/mL concentration; *n* = 8 (for EZ4U and Calcein-AM) and 20 (for Congo red) wells per group. The error bars present the S.E.M. For statistical analysis, one-way ANOVA was used followed by the Bonferroni post hoc test, and the level of significance were p*** ≤ 0.001 (*- the significant rate of deviation from the non-toxic and non-aggregated forms /Aβ 1–28, 1–42 S1, 1–42 S2, PrPC/ in both viability and Congo red assays)

In our study, we used eleven different peptides and proteins, with some of them being accepted as neurotoxic aggregates in neurodegenerative diseases (Fig. 2). To test and confirm the toxic effect of these aggregated peptides/proteins, we used a differentiated SH-SY5Y human neuroblastoma cell model, based on our previous works [7, 8]. The CRC-specific EZ4U and cytoplasmic enzyme activity-sensitive Calcein-AM assays were used to test the potential effect of the various aggregates. The time-dependent differences between the respective aggregate solutions incubated for 3 h and 3 days were measured with CR spectrophotometric assay [8, 19]. The data demonstrated an inverse correlation between CR-binding property and cellular toxicity of aggregates. The Aβ1–28, the scrambled isoforms Aβ1–42 S1 and S2, and PrPC demonstrated low affinity to CR and were not toxic to SH-SY5Y cells, accordingly.

**Fig. 3:**
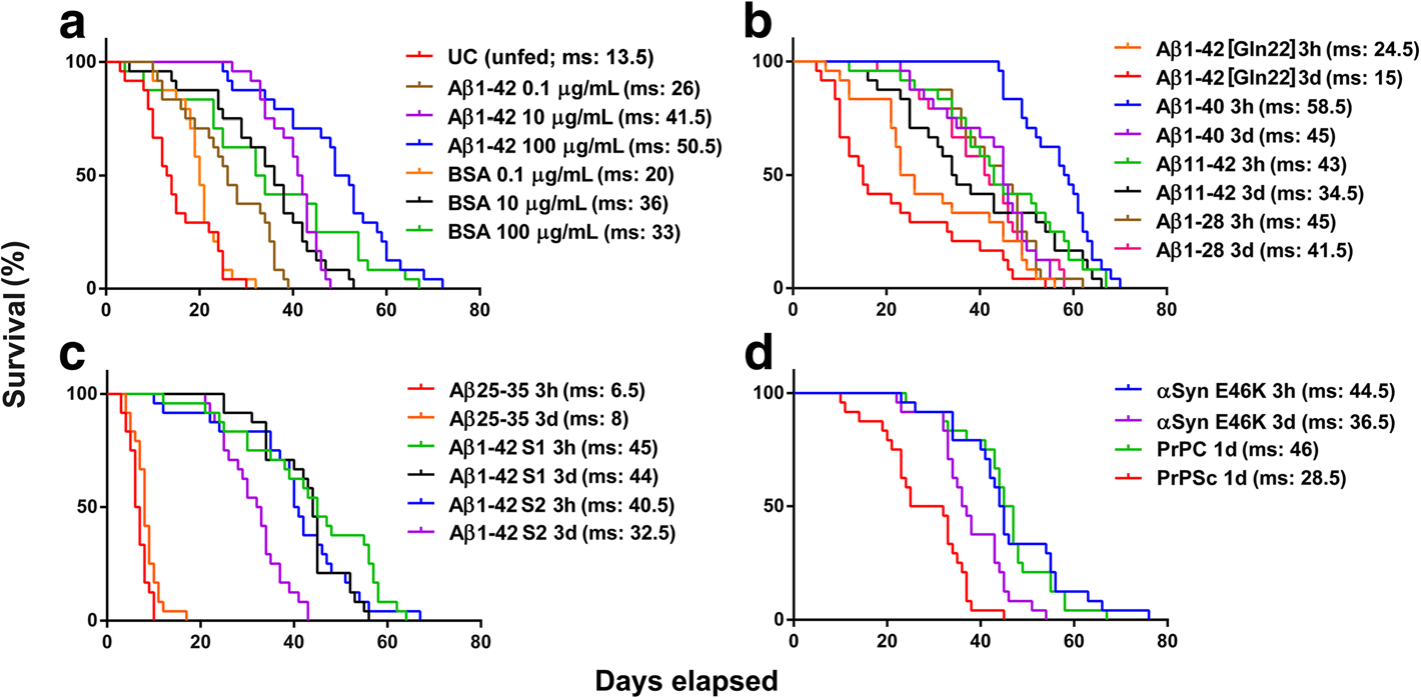
The effect of different neurodegeneration-related aggregates on the survival of *P. acuticornis.* The Kaplan-Meier curves demonstrate the survival of rotifers (n = 24 one-housed individuals per sample type; aggregation time: 3 h = 3 h and 3d = 3 days). **a** *Dose dependency*: the effect of 3 h–aggregated Aβ1–42 and BSA were tested in three different concentrations. This neurotoxic peptide as well as the non-toxic protein served as nutriments to rotifers. The survival was dose-dependent (0.1, 10, and 100 μg/mL) and was significantly higher in both type of treatments compared to the untreated control group (UC; *p* < 0.001; log-rank test). **b** *Natural Aβ isoforms:* we tested four natural variants of the Aβ peptide (1–42 [Gln22], 1–40, 11–42 and 1–28) on rotifers in two different aggregated forms (3 h and 3d). All types of natural Aβs had a positive effect on the survival of the treated rotifers compared to the UC (**a**). **c** *Artificial Aβ isoforms:* the rotifers were able to use both scrambled forms of Aβ1–42 (S1 and S2) as nutrients. As a unique exception in the series, Aβ25–35 was toxic to the rotifers, decreasing their survival as compared to the UC (**a**). All three types of artificial Aβs were measured in 3 h- and 3d–aggregated forms. **d** *Non-Aβ proteins:* the animals treated with alpha-synuclein (α-Syn, type E46K; in 3 h- and 3d–aggregated forms) and the cellular (PrPC) or ‘scrapie’ (PrPSc) forms of prion protein (1d = aggregated for 1 day) also showed a significantly longer lifespan compared to the UC (**a**). Each protein was used in 100 μg/mL concentration. The significance of log-rank (Mantel-Cox) test was *p* < 0.0001 in the statistical analyses in panels **b-d**. Median survival (ms) values in days are presented in the graphs. The statistical analysis of survival was performed by GraphPad Prism 7.0b

In our next experiment, we examined whether the *P. acuticornis* is capable of catabolizing other neurotoxic aggregates as well (Fig. 3). To investigate the dose-dependency of the effect of Aβ1–42 on rotifers, we used three different treatment concentrations (0.1, 10, and 100 μg/mL), comparing the results with those of groups treated with equivalent concentrations of bovine serum albumin (BSA). Interestingly, we observed the highest median survival in the case of 100 μg/mL Aβ1–42 treatment (Fig. 3a). We found that almost all Aβ peptide forms tested (Aβ1–42, Aβ1–42 [Gln22], Aβ1–40, Aβ11–42, Aβ1–28, Aβ1–42 S1; Aβ1–42 S2) were favourable nutrient sources, contrasting with Aβ25–35, which proved to be toxic to the rotifers (Fig. 3 b-c). This ‘short’ type of Aβ has a relatively low molecular weight (1060.27 g/mol) compared to the longer Aβ peptides (e.g., Aβ1–42), resulting in higher molar concentration (94.3 μM) in a 100-μg/mL dose. We tested this artificial Aβ aggregate in a dose of 10 μg/mL as well, and we found it also significantly toxic (NML 58 ± 5.8%; *p* ≤ 0.01) when analysing the survival of the treated group compared to that of unfed controls. In addition to various Aβ isoforms, *P. acuticornis* was also able to catabolize one of the most aggregation-prone forms of α-Syn (type E46K) as well as the physiological (PrPC) and pathological forms (PrPSc) of prion protein (Fig. 3d). The Kaplan-Meier survival curves demonstrate significant differences in all treatment groups compared to the respective untreated control curve (Fig. 3a).

**Fig. 4:**
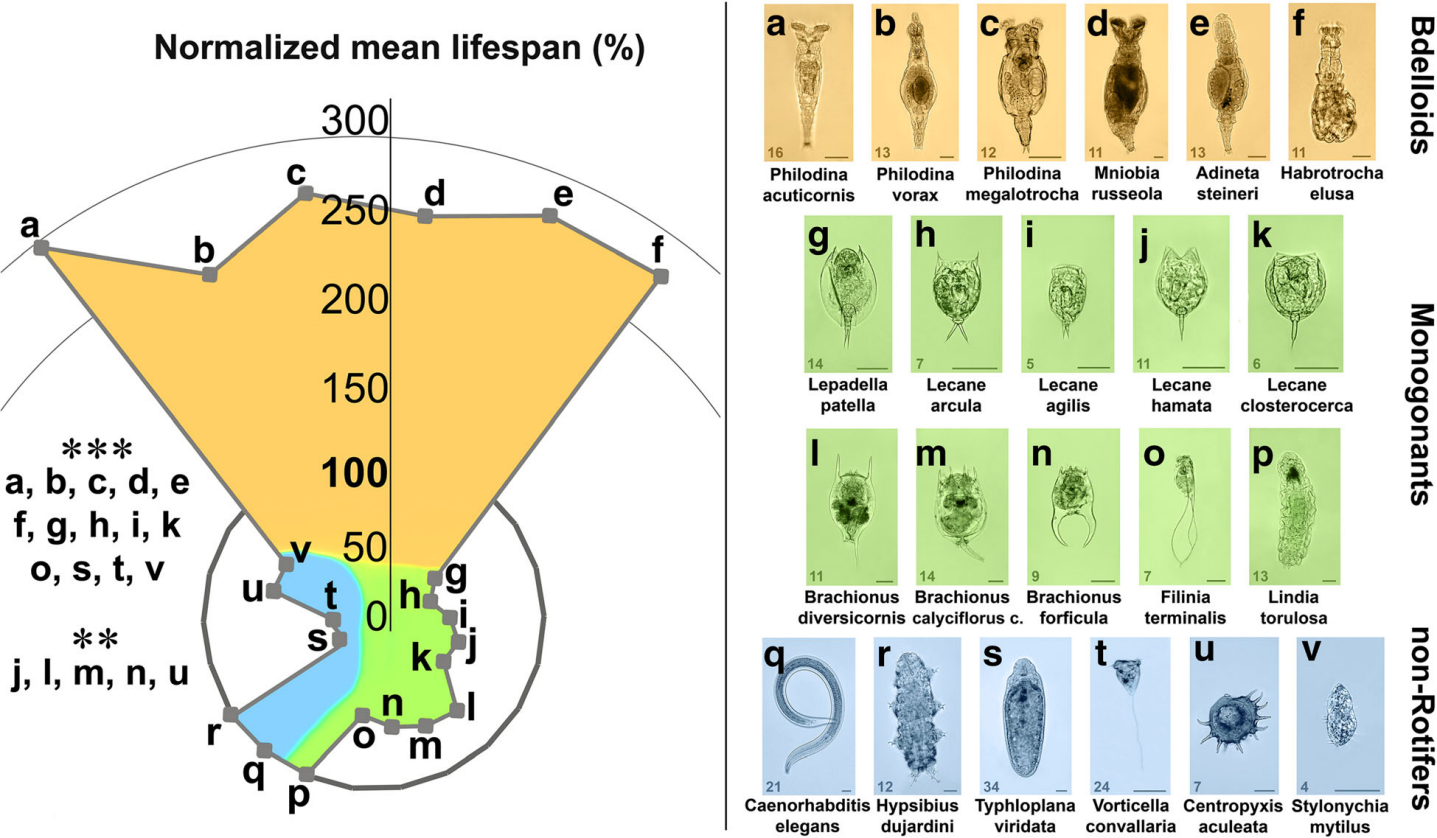
The effect of Aβ1–42 on the normalized mean lifespan of different microscopic species. To investigate the universality of the ability to catabolise 3 h–aggregated Aβ1–42 in one-housed entities, we tested 6 Bdelloids (**a-f**), 10 Monogonants (**g-p**), and 6 non-Rotifers (**q-v**). The data were normalized in percent, where the mean survival of the untreated starved group of the respective species were regarded as 100%. Only bdelloids demonstrated significantly longer lifespan compared to their starved controls after Aβ1–42 treatment. For monogonant rotifers, Aβ1–42 was either toxic (**g-o**) or ineffective (**p**) compared to their untreated controls. A similar effect was detected in non-rotifer species, where the aggregated peptide likewise either had no effect (**q** and **r**) or was toxic (**s-v**). The number in the bottom left corner of the images represents the measured real mean survival lifespan (in days) of the untreated starved control individuals. The concentration of Aβ1–42 was 100 μg/mL n = 30 one-housed individuals per group. For statistical analysis, one-way ANOVA was used followed by the Bonferroni post hoc test, and the levels of significance were p** ≤ 0.01 and p*** ≤ 0.001 (*- significant difference from the untreated starved control). Scale bars represent 50 μm

To investigate the universal capability of one-housed microscopic entities to catabolise Aβ1–42 (aggregated for 3 h before treatment; Fig. 4), we examined 22 species with different phylogenetic backgrounds. These species included bdelloid rotifers: *P. acuticornis, P. vorax, P. megalotrocha, M. russeola, A. steineri, H. elusa*; monogonant rotifers: *L. patella, L. arcula, L. agilis, L. hamate, L. closterocerca, B. diversicornis, B. calyciflorus calyciflorus, B. forficula, F. terminalis, L. torulosa*; non-Rotifers: *C. elegans, H. dujardini, T. viridata, V. convallaria, C. aculeate, S. mytilus*. In order to provide adequate basis for comparison, we used an NML where the mean lifespan of the unfed (starved) control group within the respective species were regarded as 100% (contrasting with the comparisons in Fig. 1 where 100% was the mean lifespan of the normally-fed group). From the all examined species, bdelloids (Fig. 4a-f) were the only ones that demonstrated significantly longer NMLs (240–290%) compared to their unfed (starved) controls (100%). As regards other species, Aβ1–42 was either toxic (Fig. 4g-o of monogonants and Fig. 4s-v of non-rotifers) or had no effect (Fig. 4p-r of both groups).

**Fig. 5:**
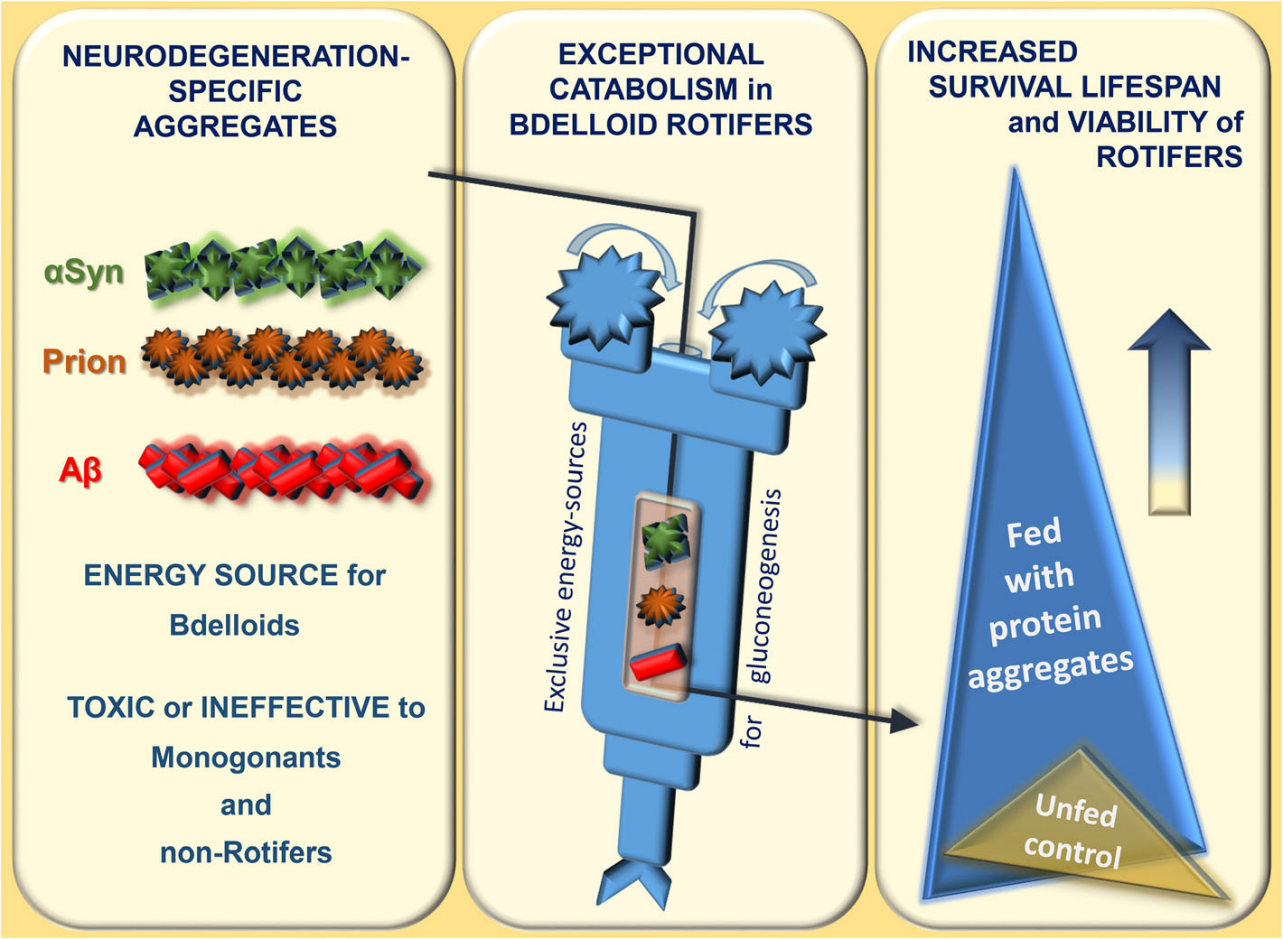
The general capability of bdelloid rotifers to catabolise neurotoxic aggregates*.* Neurodegeneration-related peptide and protein aggregates can be utilized as nutrients when given as the only potential energy source for starved bdelloid rotifer species. These agents proved to be either toxic or ineffective for monogonant and non-rotifer species. A currently unidentified mechanism in bdelloid rotifers is hypothesized to enable the utilization of amino acids derived from the degraded aggregates for gluconeogenesis. Compared to the unfed (starved) controls, the bdelloid rotifers treated with aggregates (‘fed’) demonstrate increased survival and viability

The results strongly suggest that *P. acuticornis* can catabolize various form of toxic and non-toxic Aβs, α-Syn, and prion aggregates and that the investigated bdelloid rotifer species are also able to use the neurotoxic Aβ1–42 aggregates as nutrients (ad libitum). This agent is either toxic or has no effect on the lifespan of other microinvertebrates examined, suggesting that the catabolism of aggregates with proteinopathy is an exceptional property of bdelloid rotifers (Fig. 5).

## Discussion

The formation and accumulation of aggregated proteins is a central feature of several neurodegenerative diseases. The age-related dyshomeostasis promotes the aggregation of misfolded proteins. Neurodegeneration is associated with the resistance of various aggregated peptides and proteins to enzymatic degradation [39]. In transmissible spongiform encephalopathies, the endogenous PrPC, composed of the pure protein without any presence of nucleic acid components, is converted to the pathogenic and infectious form (PrPSc) [37]. The α-Syn is a presynaptic neuronal protein, which contributes to the pathogenesis of PD via its toxic aggregation [35]. Aβ peptides can accumulate as senile plaques, which are regarded as one of the hallmark alterations in AD [2, 43].

Although the degradation of these amino acid-containing organic macromolecules has been in the focus of neuropharmaceutical science for decades, there have been no proper treatments developed or methods identified for the elimination of these toxic aggregates from living systems to date. Moreover, only a fraction of these peptides and proteins can be efficiently digested by proteolytic enzymes either in vitro or in vivo. [22]

In our study, we are the first to describe the existence of the in vivo catabolism of neurotoxic aggregates by bdelloid rotifers (e.g., *Philodina acuticornis*), as these microinvertebrates are capable of using even neurotoxic aggregates as exclusive energy and organic material sources, prolonging their lifespan. The one-housed and aggregated Aβ1–42-treated [3] *P. acuticornis* individuals have significantly longer lifespan and better viability compared to their untreated controls under complete dietary restriction in a fully isolated environment. This capability to improve (NML) or maintain (BSI, BLD, MCF and CRC) viability showed a correlation with the aggregation state (i.e., CR affinity) of Aβ1–42. The administered exogenous peptide aggregates were first localized and distributed within the digestive system of the rotifers, providing evidence of being consumed (Fig. 1). First in the literature, we demonstrated that this species have no detectable endogenous Aβ1–42 production. Since the treated *P. acuticornis* maintained its well-being, function, and redox capacity, we presume that these aggregates may serve as an obligatory energy source for gluconeogenesis in these experimental conditions. Prolonged starvation of animals causes significant physiological changes supplying glucose from amino acids by metabolism, including ketogenesis and gluconeogenesis. The main function of gluconeogenesis is to maintain the glucose level via its endogenous de novo production from non-carbohydrate substrates, such as glycerol, lactate, or glycogenic amino acids [28]. In rotifers, the resting eggs contain large amounts of non-carbohydrate substrates as obligatory sources for vital anabolic processes during dormancy and hatching via glyoxylate cycle and gluconeogenesis [10]. The bdelloids are extremely resistant to starvation, which has been shown to extend their lifespan [31], suggesting that the enzymatic machinery required for endogenous de novo glucose production may play a crucial role in their metabolism. On this basis, their observed survival in the presence of an aggregated peptide suggests its partial catabolism.

In order to assess the toxicity of the examined aggregated molecules, the same treatment agents were tested on differentiated SH-SY5Y neuroblastoma cell cultures [7, 26]. The α-Syn and PrPSc, similar to the known toxic Aβs, caused significant reduction of viability, in correlation with their aggregation state (CR affinity). Our in vitro results (Fig. 2) were in line with our a priori expectations and the academic literature [45, 56].

The catabolic capability of *P. acuticornis* was not limited to Aβ1–42 (Fig. 3). In the absence of any other potential dietary source, the presence of other neurodegeneration-related aggregates, such as natural (Fig. 3b) or artificial (Fig. 3c) Aβs; α-Syn, PrPC, or PrPSc (Fig. 3d) likewise prolonged the survival of the treated animals compared to the untreated (unfed) controls, except for Aβ25–35, which resulted in reduced survival. The median survival showed association with the aggregation state. These results together led us to the conclusion that this extraordinary catabolic activity is universal as regards almost any type of peptide and protein aggregates. In their natural habitat, bdelloids constantly have to cope with extreme fluctuations in nutrient availability [4]. Furthermore, their native food source includes particulate organic detritus, dead bacteria, algae, and protozoa, which offer a large variety of natural aggregates [50]. Therefore, the capability of these animals to metabolize almost every type of aggregated peptides and proteins might be an evolutionary strategy for adaption and survival.

Our aim was to describe the existence of the in vivo catabolism of neurodegeneration-related aggregates by bdelloid rotifers. The results strongly indicate that these animals can use these toxic agents as an exclusive dietary source to live and grown in an hermetically isolated environment. The available literature does not describe any other metazoan species with a similar capability. In species widely applied in the animal modelling of AD, such as *Caenorhabditis elegans, Drosophila melanogaster*, *Danio rerio,* or rodents, the Aβ1–42 are toxic [11, 20, 27, 41]. Based on our experiments (Fig. 4), the Aβ1–42 aggregates were also toxic or ineffective to the other microscopic entities tested (monogonant rotifers and non-rotifers). The universal capability of bdelloid rotifers to catabolise peptide and protein aggregates (including such that are known to be neurotoxic) implied that the hypothetic catabolic pathway might consist of degrading enzyme(s), their possible cofactors, and/or anti-aggregation compounds. The comprehensive identification of the molecular background of this unique phenomenon requires further analyses.

## Conclusion

In summary (Fig. 5), we present findings indicating the exceptional capability of the investigated bdelloids to catabolise neurotoxic aggregates in vivo, which is to our knowledge unprecedented in the literature regarding any multicellular animals. In our experimental system, only amino acids could be used for gluconeogenesis by the rotifers; therefore, the aggregates applied represented the only material to be catabolised in the absence of any other food source in this isolated environment. The one-housed *P. acuticornis* individuals maintain their normal phenotypic characteristics and demonstrate significant activity during their life, which require constant energy production. The prolonged lifetime in the presence of (almost all types of) Aβs, α-Syn, PrPC or PrPSc suggests the universal catabolic capability of our model species as regards a wide range of peptide or protein aggregates.

The understanding of the unique catabolic capability of bdelloid rotifers, including *P. acuticornis,* on neurotoxic aggregates in an isolated environment without any other energy source available may provide the basis of a novel therapeutic approach (identification of key molecules and metabolic pathways) in neurodegenerative proteinopathies.

## Abbreviations

α-Syn: Alpha-synuclein
AD: Alzheimer’s disease Au-Aβ Gold-tagged beta-amyloid
Aβ: Beta-amyloid
BLD: Bright light disturbance
BSA: Bovine serum albumin BSI Body size index CR Congo red
CRC: Cellular reduction capacity
CSF: Cerebrospinal fluid
DW: Distilled water
EtOH: Ethanol
IDE: Insulin-degrading enzyme
MCF: Mastax contraction frequency
NEP: Neprilysin NML Normalized mean lifespan PD Parkinson’s disease PrPC Normal cellular prion protein PrPSc Pathogenic prion protein ‘scrapie’ SEM Scanning electron microscope UC Untreated control
